# Surface Plasmon Enhanced Fluorescence Temperature Mapping of Aluminum Nanoparticle Heated by Laser

**DOI:** 10.3390/s21051585

**Published:** 2021-02-24

**Authors:** Naadaa Zakiyyan, Charles M. Darr, Biyan Chen, Cherian Mathai, Keshab Gangopadhyay, Jacob McFarland, Shubhra Gangopadhyay, Matthew R. Maschmann

**Affiliations:** 1 Department of Electrical Engineering and Computer Science, University of Missouri, Columbia, MO 65211, USA; ngzakiyyan@mail.missouri.edu (N.Z.); DarrCM@missouri.edu (C.M.D.); bcn69@mail.missouri.edu (B.C.); mathaic@missouri.edu (C.M.); gangopadhyayk@missouri.edu (K.G.); gangopadhyays@missouri.edu (S.G.); 2 J. Mike Walker Department of Mechanical Engineering, Texas A&M University, College Station, TX 77843, USA; mcfarlandja@tamu.edu; 3 Department of Mechanical and Aerospace Engineering, University of Missouri, Columbia, MO 65211, USA

**Keywords:** nanothermography, fluorescence, plasmonic, aluminum nanoparticle

## Abstract

Partially aggregated Rhodamine 6G (R6G) dye is used as a lights-on temperature sensor to analyze the spatiotemporal heating of aluminum nanoparticles (Al NPs) embedded within a tetrafluoroethylene, hexafluoropropylene, and vinylidene fluoride (THV) fluoropolymer matrix. The embedded Al NPs were photothermally heated using an IR laser, and the fluorescent intensity of the embedded dye was monitored in real time using an optical microscope. A plasmonic grating substrate enhanced the florescence intensity of the dye while increasing the optical resolution and heating rate of Al NPs. The fluorescence intensity was converted to temperature maps via controlled calibration. The experimental temperature profiles were used to determine the Al NP heat generation rate. Partially aggregated R6G dyes, combined with the optical benefits of a plasmonic grating, offered robust temperature sensing with sub-micron spatial resolution and temperature resolution on the order of 0.2 °C.

## 1. Introduction

Understanding thermal transport between constituents of energetic nanocomposites containing aluminum nanoparticles (Al NPs) is essential to the design and implementation of more efficient energetic formulations. Nanocomposites incorporating Al NPs are among some of the most widely used solid-state fuels due to the natural abundance of aluminum and high exothermicity of oxidation reactions involving aluminum. In particular, the interaction of Al NPs with reactive fluoropolymer films has attracted significant interest as fluoropolymers may be cast onto a diverse set of substrates and are compatible with additive manufacturing [1,2,3,4,5]. The reaction properties and mechanisms of energetic fluoropolymer films have been widely studied by measuring the reaction temperature evolution to understand thermal characteristics and heating kinetics [6,7,8]. Recently, we reported an in situ study of isolated fuel nanoparticles reactions by photothermal heating on a plasmonic grating platform to understand differences in the behavior of Al NP reactions with fluoropolymer and metal oxide constructs [9,10]. Despite numerous recent advances, many fundamental aspects of energy flow and reaction mechanisms of nanoenergetic materials are poorly understood, in part, due to experimental limitations in high-resolution thermal sensing.

Thermometry at sub-micron length scales has found application in microelectronics [11,12,13], microfluidics [14,15,16], and nanomedicine [17,18,19]. Enhancing the spatiotemporal resolution of temperature sensing techniques further advances the application of a fundamental heat transfer mechanism for small and rapidly heated particles. Current nanothermometry techniques include contact thermometry, including scanning thermal microscopy [20,21,22] and micro-thermocouple devices [23]. Non-contact thermometry techniques that elucidate temperature changes noninvasively are particularly attractive, including infrared thermography [24,25], quantum dot-based fluorescence thermography [26,27,28,29], and organic dye-based luminescence or fluorescence thermography [30,31,32,33]. Fluorescence thermography, in particular, offers high detection sensitivity and spatial resolution limited only by the diffraction limit on the order of a few hundred nanometers. Further, the fluorescence thermography technique is compatible with a diverse range of experimental applications, provides in situ spatial temperature maps, and results may be independent of the illumination source [34].

Numerous fluorescence-based experiments have demonstrated the utility of fluorescence thermography. Lanthanide-based dyes undergo upconversion following excitation in the near-infrared and resist photobleaching and photoblinking [35,36]. In particular, Europium-doped materials are well-suited to monitor temperatures up to 1300 K [37,38]. However, lanthanide-doped nanoparticles are frequently micron-scale, limiting their use in high-resolution applications, and offer low luminescence efficiency or quantum yield comparing to organic dyes. Several classes of organic dyes, including rhodamines, fluorescein, and triarylboron, have been used for temperature sensing [39,40,41]. These dyes may be used for temperature detection in aqueous environments such as in microfluidic devices or in various solid polymers [42,43]. In particular, Rhodamine 6G (R6G) has a long history as a dye reporter with a reversible temperature-dependent response owing to thermostability to 250 °C [15,32,44] and high temporal and spatial resolutions [44,45,46]. Unfortunately, long-duration exposure to exciting radiation may lead to irreversible photobleaching of R6G, and the relatively low signal-to-noise ratio of R6G has been insufficient to measure fast-dynamic events and low-temperature differences, limiting use of rhodamines in nanothermometry. Recently, excitation of fluorophores involving surface plasmon resonance (SPR) coupling of the incident excitation energy at a metal-dielectric interface (e.g., Ag-Al_2_O_3_) was shown to enhance the intensity of excited dyes by a factor of 100 compared to glass [47,48,49,50,51]. The free-space SPR effect provided by a dielectric-coated metal grating may also enhance the photothermal heating rate experienced by a nanoparticle near the grating surface [9,10].

Recently, we reported an in situ photothermal heating platform using plasmonic gratings with reversible temperature-induced quenching effect [52]. R6G works particularly well in a grating system with ~400 nm pitch since the coupling angles for the wavelength ranges of both excitation and emission of R6G lie near normal angles of incidence, creating an optimal window for dye fluorescence and detection of temperature effects. In the previous report, the reversible “lights-off” quenching behavior was attributed to the low concentration of R6G dye (1 µM) used in the sol precursor to avoid aggregation of the dye in the nanocomposite film. Meanwhile, temperature-induced disaggregation has been reported in highly concentrated dye films subjected to high-temperature annealing [53]. With a highly concentrated dye film, aggregates in dimer state self-quench, significantly reducing fluorescence efficiency compared to the monomer state. Temperature-induced disaggregation would result in a “lights-on” increase in fluorescence intensity with increasing temperature. Such a scheme would be preferential to a “lights-off” nanothermograph, as it would be much easier to isolate the temperature-induced response from photobleaching and reduce background interference at high temperature.

Herein, we report a R6G aggregate-based nanothermometer built upon a plasmonic grating platform fabricated by nanoimprint soft lithography [52,54]. The nanothermography system is used to analyze the spatiotemporal thermal response of photothermally heated Al NPs embedded within a tetrafluoroethylene, hexafluoropropylene, and vinylidene fluoride (THV) polymer matrix. R6G dye molecules were dispersed at sufficient concentration to form dimer aggregates in a micron thick Al NP/THV/R6G nanocomposite film. Individual Al NPs were heated using a high-power-density laser integrated into a tabletop upright microscope with a high-resolution CMOS camera for obtaining spatiotemporal image sequences of the dye response. The time-resolved fluorescence was converted to temperature maps via controlled calibration, and the temperature-dependent response was used to calculate the volumetric heat generation rate of the Al NP.

## 2. Materials and Methods

### 2.1. Substrate Fabrication

#### 2.1.1. Grating Fabrication

Plasmonic grating substrates were fabricated using a microcontact lithography stamping process [48,50,54,55,56,57,58]. A high-definition digital video disk (HDDVD) grating structure was replicated by curing 5:1 Sylgard^®^ 184 polydimethylsiloxane (Gelest) over a halved, cleaned HDDVD for 24 h at 50 °C and 55% relative humidity. Meanwhile, glass microscope slides (Corning) were cleaned by successive bath sonication in acetone, methanol, and deionized water and dried under flowing nitrogen. Cleaned slides were then soaked for 10 min in 3:1 H_2_SO_4_:H_2_O_2_ (Piranha solution), washed twice and rinsed in fresh deionized water, and dried under flowing nitrogen. Polymer ink consisting of 3% *w*/*w* GR650F polymethylsilsequioxane (Techneglas) in ethanol was spun-cast onto the stamp at 3000 rpm for 30 s and pressed onto the cleaned glass slide. The gratings were then vapor-treated with 1:1 3-aminopropyltriethoxysilane (APTES) in ethanol, pre-baked at 60 °C for 3 h, and baked at 400 °C for 1 h. A 5 nm titanium adhesion layer was added before depositing 100 nm silver (Ag) by RF sputtering and a 10 nm protective layer of alumina (Al_2_O_3_) using atomic layer deposition. Identical to previous reports [54,56], the expected topography (400 nm pitch and 60 nm peak-to-valley height) was confirmed by atomic force microscopy prior to nanocomposite film deposition.

#### 2.1.2. Nanocomposite Fabrication

**Figure 1:**
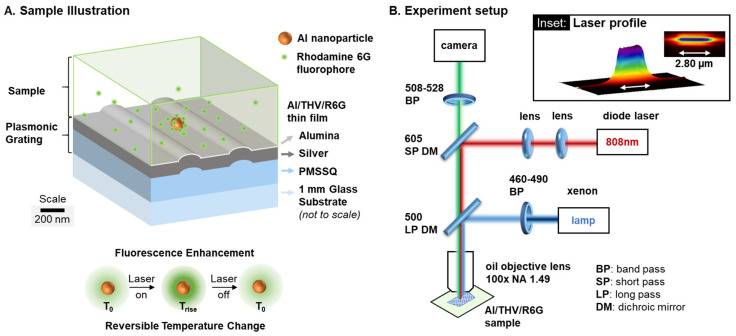
(**A**) Schematic of an energetic nanocomposite thin film deposited on a plasmonic grating substrate and (**B**) Schematic of experimental setup for in situ photothermal heating of a single nanoparticle in fluorescence-temperature measurement incorporating laser, microscope, and camera systems. (Inset) Laser profile as focused to deliver energy specifically to the aluminum nanoparticles (Al NPs).

Aluminum nanoparticles (Al NP) with 120 nm average diameter were purchased from Novacentrix (Austin, TX, USA). The aluminum nanoparticles had a core-shell structure consisting of a metal aluminum core and an Al_2_O_3_ shell of 2–3 nm as a passivation layer to protect the reactive aluminum content. In the current work, the specific oxide thickness did not play a critical role in the results; however, the shell thickness is important when reacting the Al NPs with oxidizers. Rhodamine 6G (R6G) was purchased from Exciton (Dayton, OH, USA) and THV 220A fluoropolymer with glass transition temperature (*T_G_*) of 26 °C and melting point (*T_M_*) of 120 °C from 3M Company (St. Paul, MN, USA). Al NPs and THV were separately dispersed in acetone with starting concentrations of 1 mg/mL and 60 mg/mL, respectively, and sonicated for 3 h. R6G stock solution was prepared by adding 10 mM R6G in 2-propanol (IPA) and sonicating 10 min. The constituent materials were then mixed to achieve a final concentration of 0.25 mg/mL Al NPs, 50 mg/mL THV, and 100 μM R6G, respectively, and sonicated for 2 h to ensure a well-dispersed mixture. Immediately after sonication, a 0.5 mL aliquot of the mixture was spin-coated at 3000 rpm for 30 s onto a plasmonic grating substrate produced as described above. A relatively low (0.1% *w*/*w*) ratio of Al NP to THV resulted in sparsely distributed particles within a relatively thick (~1 µm) THV matrix. The film was cured in a saturated acetone vapor environment for 4 h to improve film uniformity and smoothness [10], then dried in a vacuum oven at 60 °C for 1 h to remove any remaining solvent. The final cured nanocomposite thin film retains the low ratio of Al NP:THV, but dye concentration increases to ~4 mM due to volume reduction from solvent evaporation (Figure 1A).

### 2.2. Fluorescence Thermography Study

#### 2.2.1. Experiment Apparatus

Laser heating experiments to evaluate temperature-dependent fluorescence require the ability to collect fluorescence image sequences during laser heating, focus on specific Al NPs within the nanocomposite, and align the laser in the optical train. We designed a custom optical setup to accommodate these requirements (Figure 1B and Figure S1), which includes (i) a laser mount with focusing lenses and micropositioner, (ii) an optical filter set for beam formation and fluorescence excitation and emission, and (iii) a laser synchronization driver. A 300 mW-rated near-infrared 808 nm diode laser was chosen for the heating source since the high absorption efficiency of Al NPs in the near-infrared regime is thought to support photothermal heating by plasmonic coupling and dielectric loss [59]. Meanwhile, the 808 nm laser light will couple weakly to the silver grating at a 0° angle of incidence (i.e., normal incidence), reducing the direct heating of the grating significantly with respect to visible range lasers [52]. The laser produces a beam with elliptical Gaussian profile, which was focused by the lenses and 600 nm short-pass dichroic mirror to a beam of 2.8 µm long × 0.20 µm wide at full width half maximum on the substrate (Figure 1B, inset).

The optical filter setup is arranged to enable both fluorescence excitation and laser light to be delivered through the optical train to the sample while simultaneously screening these wavelengths from the imaging camera. Proper screening was achieved by using a pair of dichroic mirrors, including: (i) a short-pass mirror with 605 nm cut-off to reflect the 808 nm laser through the objective lens toward the sample and (ii) a long-pass mirror with 500 nm cut-off to both reflect the excitation light and pass the laser to the sample. The excitation filter (460–490 nm) and 500 nm long-pass dichroic mirror were placed in a standard filter cube slot in the microscope. The 605 nm short-pass dichroic mirror and a 508–528 nm band-pass emission filter were placed in a second filter cube and loaded in a 3D-printed adapter between the microscope proper and the camera.

#### 2.2.2. Temperature-Intensity Calibration

The temperature-dependent fluorescence spectra of R6G embedded in the nanocomposite matrix was characterized using a BioTek^TM^ Synergy^TM^ H4 Hybrid microplate reader. A xenon white light flash lamp was cut to 480 ± 9 nm excitation band and emission screened from 500–700 nm. The microplate reader was programmed to first heat the sample to 30 °C, wait for 15 min to achieve steady state, measure the spectrum, and then repeat in 5 °C increments up to 65 °C, the instrument maximum. Finally, the sample was cooled to 30 °C and measured a final time.

**Figure 2:**
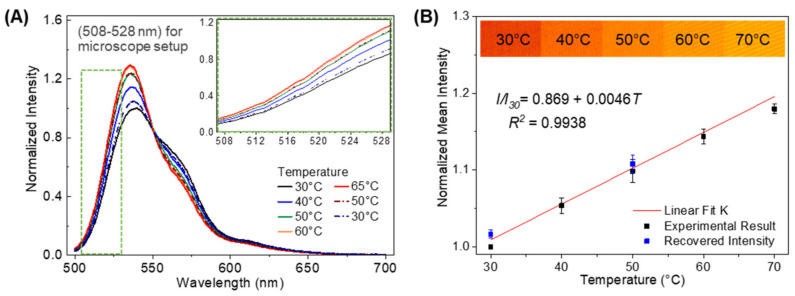
(**A**) Fluorescence emission spectra of rhodamine 6G (R6G) as a function of temperature measured using the microplate reader; solid lines represent spectra during heating cycle while dot-dash lines represent spectra during cooling. The green boxed region and inset are the 508–528 nm range selected for the imaging experiments. (**B**) Averaged R6G fluorescence micrograph pixel intensities as a function of temperature captured through 508–528 nm bandpass filter and normalized to the reference intensity at 30 °C. Theine represents linear fit with equation and associated R^2^ in plot. (Inset) False-colored fluorescence micrographs demonstrating intensity changes with increasing temperature.

The relationship between temperature and fluorescence intensity was calibrated based upon the lower wavelength/higher energy component of the R6G fluorescence spectrum, namely, the range that experiences the highest relative increase in intensity with increasing temperature (Figure 2A). To accommodate this also in the microscope, we designed a custom fluorescence filter cube that includes 460–490 nm band-pass excitation filter, 500 nm dichroic mirror, and 508–528 nm band-pass emission filter (Figure 1B). The sample was placed under the microscope objective on a closed-loop heating stage with an embedded thermocouple. The stage was slowly heated from 30 and 70 °C in 10 °C increments. After a dwell time of 15 min to achieve steady state, several images were acquired from different areas of the sample at each temperature. Fluorescence intensity was extracted from the images using ImageJ software for later conversion to temperature maps [60].

### 2.3. Fluorescence Thermography Experiment

#### 2.3.1. Laser Power Calibration

The power output of the laser was measured to obtain the irradiated power delivered to the nanoparticle through the optical train. A Newport 843-R optical power meter measured the power output of the 300-mW rated diode laser. The measured laser power was 285 mW denoting the initial power input to the experiment setup. Then, the power meter was placed in line with the 100× objective lens at the end of the optical train. After losses from the optics, laser filters, and dichroic mirrors, the laser power delivered to the sample was 0.1 mW. Using this measured laser power and the laser dimension, the power density of the laser was approximately 17,000 W/cm^2^ at the sample surface.

#### 2.3.2. Laser Heating Experiment

Heating of single Al NP in the THV/R6G matrix was captured using an Olympus BX51WI epifluorescence microscope equipped with Hamamatsu ORCA flash 2.8 CCD camera. Images were obtained at 40 frames per second (fps) at 2.8-megapixel resolution, providing a temporal resolution of 25 ms. The imaging objective lens was an Olympus UAPON 100× oil-immersion lens with a numerical aperture of 1.49. The combination of the high-power objective lens and grating coupling effect provided subwavelength resolution (36 nm per pixel), facilitating the visualization of an individual nanoparticle [10]. ImageJ software was used to process experimental images and to convert fluorescence intensity into temperature maps. A time-averaged sequence of the three frames just prior to laser heating was collected to use as an ambient reference image to reduce inherent signal noise. The temperature conversion normalized each frame to the initial ambient temperature intensity. Each subsequent heating frame (***I***) was normalized by the initial time-averaged frame (***I*_30_**) and translated to a temperature map via pixel-by-pixel conversion.

## 3. Results and Discussion

### 3.1. Temperature-Intensity Calibration

#### 3.1.1. Temperature-Dependent Spectral Properties

The fluorescence spectra of R6G in the Al NP-R6G-THV nanocomposite taken in the microplate reader is given in Figure 2A. The “initial condition” spectrum acquired at 30 °C showed a dominant primary peak at 540 nm and a shoulder peak at ~560 nm. These peak locations were blueshifted by about 10 nm with respect to the expected peak locations for dye aggregates in either solution [61,62,63] or solid thin films [64]. We attribute the blueshifted fluorescence emission to the well-documented surface plasmon-coupled emission (SPCE) effect over plasmonic gratings [57,59], whereby the excited state energy of the fluorophore was transferred nonradiatively back to the grating prior to radiative emission by scattering from the grating itself. This process reduced the energy lost to internal conversion within the dye prior to radiative emission and produced a blueshift regardless of other effects. Considering the plasmon-induced blueshift, we associate the 540 nm peak with the R6G monomer and the 560 nm peak with a population of randomly oriented J-type dimers formed by aggregation in the THV matrix [65]. While the concentration of R6G in solution is 100 µM, solvent evaporation during spin-casting and subsequent curing and annealing steps greatly reduced the total volume of the nanocomposite matrix so that the final concentration of dye was on the order of 4 mM. The interactivity of the fluoropolymer with R6G was expected to be low, suggesting that dye molecules may have been sequestered together in pockets formed by the evaporating solvent. Combining these effects, the transition of R6G concentration from 10^−4^ M to 10^−3^ M regime was sufficient to form dimers with lower quantum efficiency, both through increased internal conversion of the J-type dimers prior to emission as well as quenching of remaining monomer units through monomer-to-dimer energy transfer [65].

Upon heating, the primary peak experienced an increase in intensity with increasing temperature, and the peak wavelength blueshifted from 540 nm at 30 °C to 535 nm at 65 °C (Figure 2A, solid lines). Meanwhile, the 560 nm shoulder peak decreased in intensity with increasing temperature. The combination of shifting and increasing primary peak intensity and decreasing shoulder intensity indicates that increased temperature promotes disaggregation of dyes, either J-dimer decoupling into individual monomers or separation of monomers beyond the energy transfer distance from adjacent dimers, allowing the recovery of monomer fluorescence emission. In samples both liquid and solid, blueshifting upon exposure to light may also have resulted from simple photobleaching, which irreversibly damages dyes and isolates remaining emissive molecules as damaged dyes are screened out of the optical system. However, the recovery of the quenched fluorescence emission state on the cooling cycle demonstrates the reversibility of the temperature-induced peak-shifting effect (Figure 2A, dash-dot lines). Moreover, the films were highly stable and reusable over several experiments with little change in spectral properties, including intensity, peak locations, and response to heating and cooling cycles, so photobleaching was not considered a significant contributor to this effect. Two potential mechanisms can explain the reversibility of heating after disaggregation. First, dye molecules participating in J-dimer aggregates may separate or experience a change in angle of association with increasing temperature, recovering quantum efficiency lost to internal conversion and reabsorption in the J-dimer state [53]. Second, disaggregation and separation are further supported by the relatively low glass transition temperature of THV (*T_g_* = 26 °C). Essentially all of the calibration spectra were collected above this temperature, so the polymer chains will become increasingly mobile with increasing temperature and time. Thus, monomers quenched by energy transfer to J-dimers or higher order aggregates may separate sufficiently to recover fluorescence emission. Both effects are likely to contribute to the overall increase in monomer fluorescence emission. Viscous deformation of THV may also contribute to the residual shift in the spectra upon cooling to 30 °C, as some dyes are permanently isolated from each other and do not return to sufficient proximity for energy transfer as the surrounding matrix relaxes to a new, more thermodynamically favored resting state. While the focus of the present work is to use the dye as a reporter for temperature, the exact processes governing the fluorescence emission of the highly concentrated state in the presence of plasmonic grating and associated temperature dependence are under further study.

#### 3.1.2. Temperature-Dependent Image Properties

Correlating the temperature-dependent effects captured by the microscope with the changes seen in the microplate reader is essential to defining the spatial characteristics of the film response during nanoparticle heating [52]. For image-based thermography, our goal was to selectively capture the “lights-on” temperature-dependent increase in the primary peak seen in the spectra above. Whereas spectroscopic analysis considers wavelengths of light individually, and a ratiometric measurement between the primary peak and shoulder would be preferable, image-based thermography considers all captured wavelengths in a single pixel as one data point for that region in space. A decrease in photons of wavelengths represented in the shoulder peak would also detract from the total photons captured by the camera and dampen any temperature-dependent increase in the primary peak. In other words, we must sacrifice spectral resolution in favor of spatial resolution in this experiment. Hence, a 508–528 nm bandpass emission filter was selected to screen out the shoulder peak for imaging experiments and capture only the portion of the primary peak that increases with increasing temperature (Figure 2A, inset). The resulting fluorescence intensities of seven distinct regions of the film without Al NPs were averaged together to produce a single calibration point for each temperature and normalized to the reference intensity at 30 °C (Figure 2B). The relatively small standard deviation about the mean at each point demonstrates the temperature uniformity throughout the entire field-of-view of the sample. A clear linear relationship was observed between film temperature and the fluorescence emission intensity when the film was heated from 30 °C to70 °C. The fluorescence image intensity increases ~200 (a.u.) over the increasing temperature of 40 °C, and the calibrated temperature resolution is on the order of 0.2 °C. A first-order linear approximation was fit to the normalized data (*R*^2^ = 0.994) and given as: $\frac{I}{I_{30}}=0.869+0.0046T$, where *I* is the fluorescence intensity at arbitrary temperature, *I*_30_ is the reference fluorescence intensity at 30 °C, and *T* is the temperature. Solving for *T*, we find that

(1) $$\;T\;=\;217.39\;*\;\frac{\;I}{I_{30}}\;-\;188.9$$

After one heating cycle, the sample was slowly cooled and the intensities at 50 °C and 30 °C were captured to demonstrate the reversibility of the temperature-dependent response (Figure 2B, blue points). These two points were chosen as settling to steady state during cooling took significant time after heating with the Peltier device as the grating and glass substrate continued to contribute heat to the R6G-THV system after the Peltier had been cooled. Critically, the fluorescence was found to recover after cycling back to 30 °C with no statistically significant difference in intensity. The reversibility and repeatability of the intensity increase means that a single particle might be used for multiple experiments, especially those exploring the effect of increasing laser fluence on heating and heat dissipation.

The temperature resolution and response sensitivity to changes in temperature are of significant importance as these factors determine how reliably we can observe changes in temperature due to laser heating. Temperature resolution (unit of °C) measures the smallest change in temperature in respect to the change in fluorescence indicator, whereas the relative or response sensitivity (unit of %/°C) is the percentage of the change in the fluorescence indicator to itself in respect to change in temperature. Herein, we have achieved temperature resolution of 0.2 °C and response sensitivity of −0.4%/°C. These values compare well to those of prior reports using molecular dyes as the temperature reporter. As stated above, molecular dyes in the rhodamine family have been widely researched for their thermometry applications. One of the most representative examples is the use of rhodamine B for measurement of fluid temperature in microfluidic systems [14]. The technique measures the temperature distribution of microfluidic circuits as a consequence of Joule heating. The photoluminescence intensity and temperature response were calibrated up to 90 °C with temperature resolution ranging from 0.03–3.5 °C depending on the signal processing method. The spatial and temporal resolutions achieved were 1 µm and 33 ms, respectively. More recently, fluorescent rhodamine B-doped latex particles were developed as temperature sensing devices with reversible response [66]. The peak fluorescence intensity had a linear response to temperature changes over the range of 30−100 °C with response sensitivity of −0.93%/°C. This intracellular heat mapping with sub-micrometer resolution demonstrated a nonuniformity of temperature distribution of locally heated gold nanoparticles excited by a laser. Rhodamine derivatives have also been incorporated for super-resolution cell imaging at spatial resolution of 50 nm, although the temporal resolution was on the order of seconds [67,68]. Previously, our group reported a thermometry sensor having fluorescence intensity decreases with temperature attributed to quenching behaviors with temperature resolution of 0.14 °C and response sensitivity of −0.38%/°C [52]. Although that report demonstrated superior temporal resolution at 2 ms, the 300 nm spatial resolution was lower compared than the 36 nm/pixel achieved in this work. Additionally, the dye concentration has been altered such that fluorescence intensity increases with temperature.

### 3.2. Characterization of Al NPs Heated in R6G-THV Matrix

**Figure 3:**
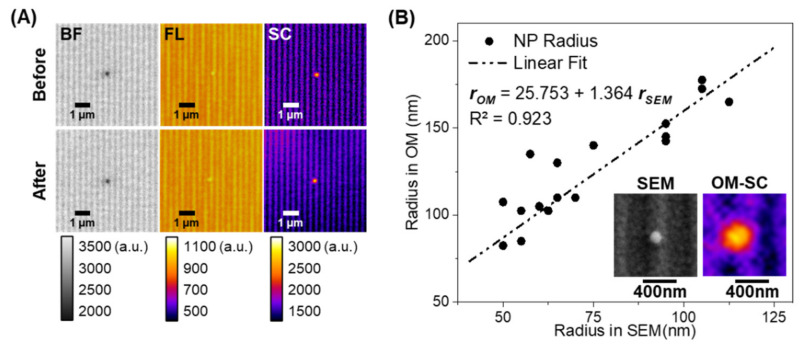
(**A**) Single Al NP imaged before and after laser-induced heating using brightfield (BF), fluorescence (FL), and polarization-based scattering (SC) modes. (Note: images are false color with intensity indicated by the intensity bars. (**B**) SEM and polarization-based scattering optical microscope images are used to calculate the ratio to measure the actual dimension of the nanoparticle. (Inset) SEM and optical microscope scattering (OM-SC) images of the same Al NP at the same relative scale.

The morphologies of Al NPs embedded in the R6G-THV matrix were characterized by brightfield, fluorescence, and scattering imaging modes before and after laser-induced heating (Figure 3A). Brightfield imaging was used to locate single Al NPs and to focus and align the laser so that the targeted Al NP was aligned to the center of the laser beam profile. The brightfield imaging mode is particularly useful for identifying single particles in the presence of R6G since brightfield interrogation uses the lowest overall light intensity, minimizing the potential for photobleaching prior to laser heating. Initially, Al NPs in brightfield mode appeared as dark spots against the bright grating background due to the relatively low reflection of light. No noticeable changes in morphology were apparent in the Al NP after heating with 808 nm laser. Previously, we reported that heating of Al NP clusters with 808 nm laser generated a greater heating rate than other wavelengths used in that work [52,59], so the absence of morphology change suggests that no permanent effects (e.g., ignition) occurred during heating with the 808 nm laser at the current laser fluence. This was confirmed by the equivalent image in scattering mode using both polarizer and analyzer at 0° with respect to the grating axis, which revealed depolarization effects due to the metallic content of Al NP. At 0°, any backscattered s-polarized light from the grating is screened out, while p-polarized light is coupled to the grating. Thus, the only remaining high intensity light to reach the camera is the scattered, depolarized light from the Al NP. Analyzing such changes in scattering intensity has been used extensively for in situ observation of laser-ignited Al NP and THV combustion [9,10]. Al NPs heated in the current experiment do not show the characteristic changes in scattering intensity brought about by restructuring and oxidation of the aluminum during laser-induced combustion. This result is critical as the subsequent studies demonstrate the heat dissipation of Al NPs to their environment as a result of heating alone rather than temperature increases due to more catastrophic laser-induced combustion events.

In fluorescence mode, R6G serves as a reporter dye with the initial temperature-independent pixel intensity correlating with the local electric field due to the plasmonic effect and those mentioned in the previous section on calibration. Both R6G excitation and emission spectra overlap strongly with the surface plasmon resonance coupling peaks of the grating at near-normal incidence, resulting in enhanced fluorescence intensity of the fluorophores and enhanced capture through directed emission toward the photodetector (i.e., camera) [52]. Excitation may be enhanced via plasmon resonance energy transfer (PRET), while emission is enhanced due to aforementioned SPCE [50,69,70]. The grating lines are apparent in the fluorescence micrograph as the plasmon-induced electric field varies between the peaks and valleys of the grating. Notably, the fluorescence of R6G localized near the Al NPs is elevated with respect to the surrounding bulk. Previously, we reported that large clusters of Al NPs appeared as dark regions in the center of a bright, fluorescent field due to the inability of the R6G dye to penetrate between the constituents of the Al NP cluster [52]. By contrast, the particles in Figure 3A appear as bright, contiguous spots indicating R6G dye can fully coat the Al NP surface and, critically, that we have visualized a single Al NP. This is due in part to plasmonic interaction between the Al NP and the Ag grating across the small dielectric gap created by the Al_2_O_3_ layer and any R6G-THV matrix between the Al NP and grating surface. This nanogap serves as a hotspot for an electric field, which further enhances the fluorescence of dyes localized to the particle than that of dyes localized to the grating. However, this increase in initial intensity over Al NPs does not appear to impact the relative temperature-dependent response, and so, the relative intensities are still governed by Equation (1), given above. This feature is important to distinguish between single Al NPs and Al NP clusters, allowing us to classify particles by cluster size and isolate classes of particles for further study.

Accurate measurement of the Al NP radius is critical to determining the volumetric energy generation rate of photothermally heated Al NPs. Figure 3B compares the measured radii of several Al NPs, determined from scattering images in the optical microscope with the ground truth radii determined for the same particles by FEI Quanta 600 FEG scanning electron microscope (SEM). The observed dimension of nanoscale Al NPs in the optical microscope is slightly larger than the reported dimension due to the diffraction limit and the size-dependent absorption and scattering cross-sections of Al NPs, which produce a cone of light emitting from the particle. The conversion between SEM images to optical microscope scattering images can be fit to a linear relationship with R^2^ = 0.923 (Figure 3B text).

### 3.3. Laser-induced Photothermal Heating

**Figure 4:**
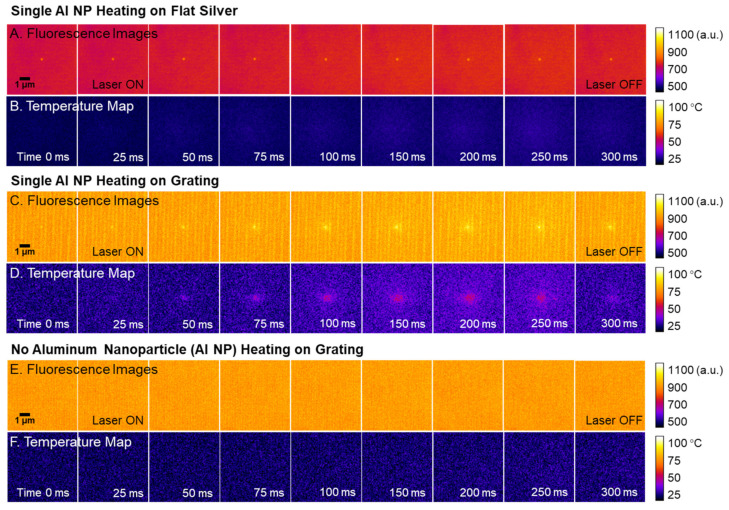
The time sequence of fluorescence intensity and temperature during laser irradiation was shown in the false colored images. Heating of single nanoparticle embedded in a 1 μm Al/THV/R6G film and supported on (**A**,**B**) flat silver and (**C**,**D**) plasmonic grating substrates indicating rapid localized heating followed by rapid cooling after the laser is turned off. (**E**,**F**) The laser also irradiated Al/THV/R6G film on a grating substrate with the absence of Al NP.

The mechanism of Al NP photothermal heating occurs via absorption of incident photons at the surface of the nanoparticle [71], which excite electrons and generate phonons through electron-lattice relaxation processes [72]. Thermal conduction spreads the heat within the nanoparticle and to the surrounding THV film. Figure 4 shows sample sequential fluorescence micrographs of the Al NP-R6G-THV nanocomposite in different platform and experimental conditions to demonstrate the importance of grating coupling on the elucidation of laser-induced heating. Notably, the embedded Al NPs supported on a flat silver substrate do not show an appreciable increase in temperature, as seen in the converted temperature map (Figure 4A,B).

The plasmonic grating acts as a reflective substrate and a light coupling device to generate more heat than a flat silver substrate under laser photothermal heating (Figure 4). The heating rate of Al NPs with 808 nm laser is enhanced from the plasmonic coupling, which falls within the broad Al absorption spectrum. Absorption spectra consist of the combination of plasmon resonance and interband transitions [73,74]. The 808 nm near-IR laser overlaps with a portion of Al absorption spectra associated with the dielectric loss [72]. The interband transitions of bulk aluminum have an intrinsic peak at 810 nm and the peak intensity grows with increasing particle diameters [75]. Below 600 nm, Al NPs can exhibit dipole, quadrupole, and octupole plasmonic resonances, of which peak wavelengths are red-shifted as the diameter increases [76]. The better confinement of light at these lower wavelengths leads to higher absorption such that the 808 nm wavelength exhibits a relatively weak plasmonic coupling compared to other laser wavelengths. For instance, 446 nm blue and 632 nm red lasers have been utilized as a heating source for Al NP and show a greater electromagnetic coupling due to higher plasmonic absorption, rather than its dielectric loss [10]. The effect of the plasmonic heating leads to heating of the grating, which is undesirable for thermometry purposes. The use of the 808 nm laser was beneficial due to the drastically diminished heating of the grating substrate.

The duration of laser heating was selected to study the local temperature of the heated film surrounding the nanoparticle. In a separate experiment (Figure S2), the Al NP was exposed to laser heating for a duration of 400 ms with images obtained every 25 ms. The THV/R6G film reached a stable temperature profile at ~200 ms; hence, all experiments presented here used a laser exposure time of 250 ms. Each nanoparticle was imaged in brightfield, fluorescence, and scattering modes before and after heating. Note that the plasmonic grating is identifiable in the background of the images (Figure 4). More importantly, the isolated single nanoparticle can be readily distinguished from small Al NP clusters in scattering mode. No optical or morphological changes of Al NPs in the THV film as a consequence of heating were observed, indicating that the NP did not react with the surrounding THV. Excessive laser heating may melt the surrounding THV medium at about 120 °C, causing the particle to shift its position, which was previously reported [52]. Hence, the laser heating was maintained below this critical value to avoid any Al NP translation.

During particle heating, the fluorescent intensity of the dye-coated nanoparticle increased rapidly under laser irradiation. The surrounding THV matrix fluorescent intensity also increased in the immediate vicinity of the Al. A representative dynamic image sequence demonstrating the transient fluorescence and temperature maps is shown in Figure 4. These images represent a 6 µm × 6 µm cropped area from the raw images with a dimension of 69.8 µm × 52.4 µm (Figure S3). The temperature rise of the Al NP is apparent, and the Al NP and THV reach a steady state temperature profile at ~200 ms. Based on measurements obtained in concentric circles around the heated NP, the temperature distribution was radially symmetric, further emphasizing that no additional heat was generated from the THV medium or the plasmonic grating. When the laser heating was extinguished at 250 ms, the temperature of the THV rapidly decayed.

It is important to note that the fluorescence intensity changes were not observed in the absence of an Al NP. As an initial control experiment, the laser was irradiated on a sample area without Al NPs for 250 ms. No increase in intensity from the initial temperature was observed, indicating that the THV, which has a low optical absorption at 808 nm, and the grating substrate do not absorb the laser energy shown in Figure 4E,F. Silver is widely used in plasmonic applications due to its inherently low loss [50,77]. In contrary, aluminum, is a high loss material and not an attractive plasmonic material with interband transition located in the near-IR. These findings indicate that Al NP is the only source of laser energy absorption and the heating source to the surrounding THV medium.

### 3.4. Aluminum Nanoparticle Photothermal Heat Generation

The plasmonic grating not only enhances fluorescence excitation and emission but also the local electric field and photothermal heating experienced by a nanoparticle in close proximity to the grating. The electric field enhancement factor provided by a plasmonic grating is difficult to determine experimentally and is a complex function of NP radius, NP composition, laser wavelength and incidence angle, and placement of the NP relative to the grating ridges. The spatially resolved THV temperature profiles enabled by the dye facilitated an estimation of the THV temperature gradient and, by extension, the Al NP heating rate for each particular nanoparticle radius.

**Figure 5:**
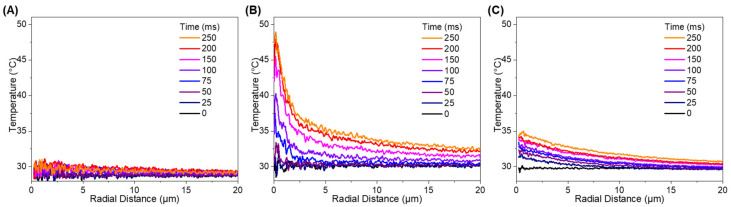
The time sequence of radial temperature profiles during laser irradiation. (**A**) The laser initially irradiated Al/THV/R6G film on a grating substrate with the absence of Al NP. Radial temperature profiles from the temperature maps of heated 120-nm Al NP supported on a (**B**) plasmonic grating and (**C**) flat silver substrates showing time-sequence before (at 0 ms) and during laser-induced heating.

For each of the experiments, the resulting fluorescence images were converted into a spatial temperature map. Scattering images were used to estimate the dimension of each Al NP using the correlation shown in Figure 3B. A representative transient THV temperature profile resulting from a single 120 nm diameter Al NP is shown in Figure 5. Radial temperature profiles were obtained by averaging the temperature map over the entire angular domain from 0–2π radians in ImageJ software. To smooth the raw data, binning was performed for each pixel with the average of its 3 × 3 pixel neighborhood. The temperature in the vicinity of the heated single Al NP increased and reached steady state with a peak temperature of 48.5 °C within 200 ms. While the region of greatest temperature was localized around the Al NP itself, the heat-affected region extended radially outward for tens of microns with time (Figure 5).

**Figure 6:**
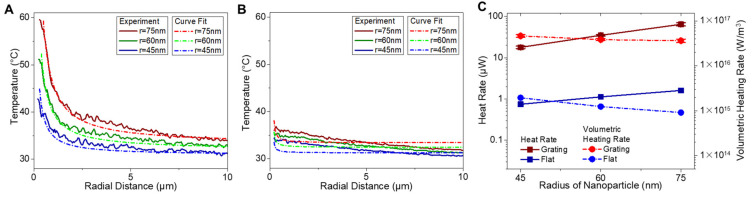
Steady state radial temperature profiles were obtained experimentally for photothermally heated Al NP with the radius of 45 nm, 60 nm, and 75 nm on the (**A**) grating and (**B**) flat silver substrate. The (**C**) heat generation rate and volumetric energy generation rate can be derived from the experimental temperature profiles.

The resulting temperature profiles for three different Al NP radii supported on a plasmonic grating and flat silver substrate are summarized in Figure 6A,B. For all experiments, the temperature profile generated on the grating substrate exceeded that observed for a similar Al NP supported on a flat silver substrate for identical laser heating parameters. The temperature profile observed for both substrates increased with Al NP radius; furthermore, the temperature difference between the grating and flat silver substrates increased with radius. As an example, the maximum THV temperature recorded using a plasmonic substrate increased from 42.7 °C to 59.8 °C as the Al NP radius increased from 45 to 75 nm. The maximum temperature observed using a flat silver substrate increased from 34.4 °C to 36.9 °C for the same Al NP radii.

The heat generation rate of the central Al NP can be estimated from the radial temperature profile of the THV [49]. For a first-order approximation, the system was treated as a homogeneous THV slab with a point heating source. The resulting temperature profile is radially symmetric such that the steady state temperature profile may be readily obtained from the heat diffusion equation. Denoted as “curve fit data” in Figure 6A,B, the temperature profile of the THV matrix was fit to an assumed solution of $T\left(r\right)=-C_{1}/r\;+\;C_{2}$, where $C_{1}$ and $C_{2}$ are the curve fit constants, and r is the radial distance from the center nanoparticle. Note that the fit was performed preferentially to the first micron of the radial distribution, as the thickness of the film, convection from the top of the THV film, and losses to the grating substrate will invalidate the spherical approximation. Nevertheless, the approximation holds relatively well for radial distances that far exceed the thickness of the film. Origin software was utilized to enable the curve fits of the experimental data. The radial temperature derivatives, namely, the heat transfer rate and the volumetric heating rate shown in Figure 6C, can readily be obtained from the approximation of experimental temperature profiles. Data points and standard deviation error bars were based on four experimental measurements for nanoparticle heating experiments on the silver grating (Figure S4).

The total Al NP heating rate may then be found from the temperature gradient of THV surrounding the NP. The NP heating rate is found using the relationship

(2) $$q=-k\left(4\pi r_{o}^{2}\right)\frac{\partial T\left(r_{o}\right)}{\partial r}$$

where *q* is the heating rate of the Al NP, *k* is the thermal conductivity of THV, and *r_o_* represents the interface between the THV and the heated Al NP [1,10,54]. From the total heating rate, the volumetric heating rate of the Al NP can be obtained by dividing the total heating rate by the volume of the Al NP. For photothermal heating on a grating substrate, the volumetric heating rate of Al NP with radii of 45, 60, and 75 nm is 4.66 × 10^16^, 3.85 × 10^16^, and 3.64 × 10^16^ W/m^3^, respectively, as summarized in Table 1. For Al NPs heated on the flat silver substrate, the volumetric heating rate was 1.95 × 10^15^, 1.24 × 10^15^, and 0.91 × 10^15^ W/m^3^, respectively. Based on these results, the heating rate of NPs due to field enhancement from the grating was between 24–40× greater than that observed for a flat silver substrate.

## 4. Conclusions

Fluorescence-based R6G dyes were used as partially aggregated, in situ temperature sensors to evaluate the photothermal heating of single Al NPs of 45 nm–75 nm diameters. A plasmonic grating platform was used to enhance the fluorescence intensity, optical resolution, and heating intensity. The dye concentration was designed to achieve a reversible and “lights-on” temperature-dependent behavior, likely a result of heating-induced transition of R6G dimerized aggregate into monomeric form and separation of monomers from remaining dimers beyond the energy transfer distance. The R6G sensors were employed as thermal probes to determine the temperature response of photothermally heated Al NPs that were distributed within the same THV media. The fluorescence intensity profiles were converted into spatial temperature maps comprising a spatial resolution of 36 nm and a temporal resolution of 25 ms. The thermal response of Al NPs is expected to be closely related to the radius of the NPs themselves, so the Al NP radius observed by polarization-based optical scattering was calibrated to SEM images to provide a grounded truth. The experimental temperature profiles were used to estimate the photothermal energy generation rate of the Al NP. The sensitive, linear fluorescent response of the R6G dye, coupled with the fluorescence intensity enhancement provided by a plasmonic grating substrate, enabled a quantitative temperature sensing modality at a spatial resolution of less than 50 nm per pixel, even for the modest temperature differences presented in this work. This work represents a robust platform for high-resolution imaging and temperature sensing for sub-micron scale investigation.

## Figures and Tables

**Table 1:** Experimental result for nanoparticle embedded in flat silver and silver grating.

Parameters	Flat Silver			Silver Grating		
Nanoparticle Radius (nm)	45	60	75	45	60	75
Experimental Heat Rate (µW)	0.746	1.124	1.612	17.790	34.837	64.257
Experimental Volumetric Heating Rate (W/m^3^)	1.95 × 10^15^	1.24 × 10^15^	0.91 × 10^15^	4.66 × 10^16^	3.85 × 10^16^	3.64 × 10^16^

## Data Availability

Data sharing not applicable.

## Supplementary Materials

The following are available online at https://www.mdpi.com/1424-8220/21/5/1585/s1, Figure S1. A photo of the experimental setup for in-situ photothermal heating of a single nanoparticle in fluorescence-temperature measurement incorporating laser, microscope, and camera systems. (Inset) Laser profile as focused to deliver energy specifically to the Al NPs. Figure S2. Al NP was exposed to laser heating for a duration of 400 ms with images obtained every 25 ms. Only some time steps of the radial temperature profiles are presented to assist observation. The temperature rise of the Al NP is apparent, and the Al NP and THV reach a steady state temperature profile at ~200 ms–250 ms. Figure S3. False-colored fluorescence image captured before heating showing the location of the target Al NP. The inset depicts the Al NP aligned to the focus of the laser prior to laser exposure. (b) During laser heating, the fluorescence intensity was elevated due to the photothermal heating of Al NP. (c) The temperature map is obtained from the fluorescence image using the calibration equation. Intensity bars for (a) and (b) indicate the fluorescence intensity while (c) is the temperature (°C). Figure S4. Steady state radial temperature profiles were obtained experimentally. Four independent heating experiments were conducted for each nanoparticle radius: (A) 45 nm, (B) 60 nm, and (C) 75 nm.
