# Supplementary material for: Surface Plasmon Enhanced Fluorescence Temperature Mapping of Aluminum Nanoparticle Heated by Laser
Source: Sensors (Basel). 2021 Feb 24;21(5):1585. doi: 10.3390/s21051585 (PMC7956715; doi:10.3390/s21051585)
Supplement: Supplementary file 1 [file sensors-21-01585-s001.pdf]

sensors

Supplementary Material

Article

Plasmonic-Enhanced Fluorescence Temperature Mapping of Aluminum Nanoparticle Heated by Laser

Naadaa Zakiyyan[1], Charles Darr[1], Biyan Chen[1], Cherian Mathai[1], Keshab Gangopadhyay[1], Jacob McFarland[2], Shubhra Gangopadhyay[1], Matthew R. Maschmann[3,*]

1 Department of Electrical Engineering and Computer Science, University of Missouri, Columbia, MO 65211, United States
2 J. Mike Walker Department of Mechanical Engineering, Texas A&M University, College Station, Tx, 77843, United States
3 Department of Mechanical and Aerospace Engineering, University of Missouri, Columbia, MO 65211, United States
* Correspondence: MaschmannM@missouri.edu

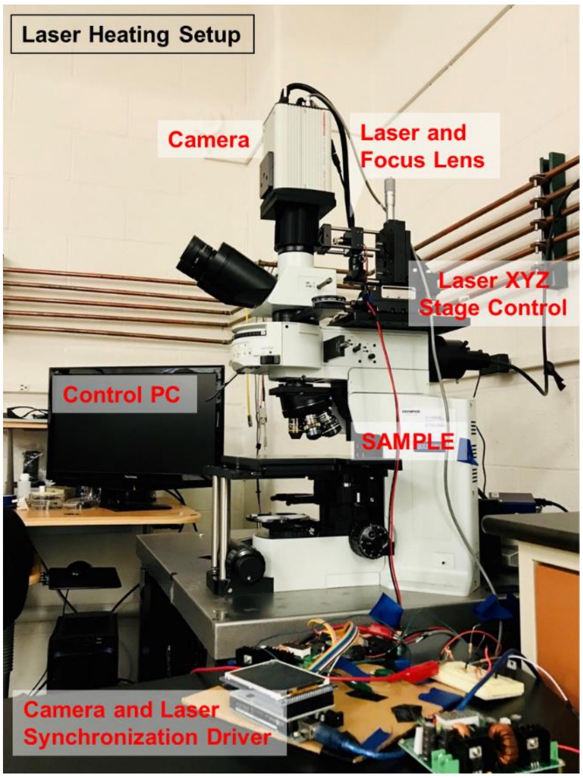

Figure S1. A photo of the experimental setup for in-situ photothermal heating of a single nanoparticle in fluorescence-temperature measurement incorporating laser, microscope, and camera systems. (Inset) Laser profile as focused to deliver energy specifically to the Al NPs.

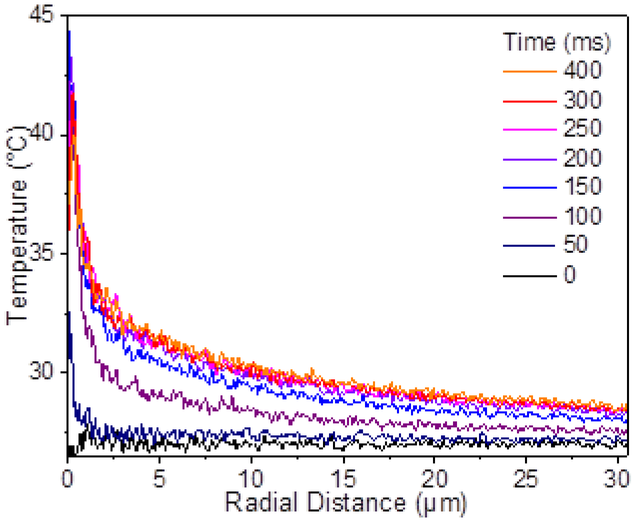

Figure S2. Al NP was exposed to laser heating for a duration of 400 ms with images obtained every 25 ms. Only some time steps of the radial temperature profiles are presented to assist observation. The temperature rise of the Al NP is apparent, and the Al NP and THV reach a steady-state temperature profile at ~200 ms - 250 ms

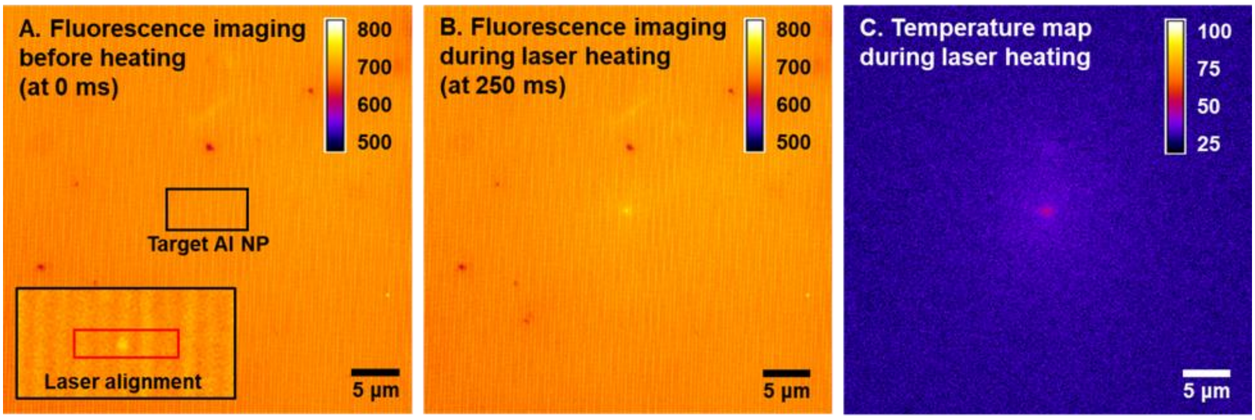

Figure S3. False-colored fluorescence image captured before heating showing the location of the target Al NP. The inset depicts the Al NP aligned to the focus of the laser prior to laser exposure. (b) During laser heating, the fluorescence intensity was elevated due to the photothermal heating of Al NP. (c) The temperature map is obtained from the fluorescence image using the calibration equation. Intensity bars for (a) and (b) indicates the fluorescence intensity while for (c) is the temperature (°C).

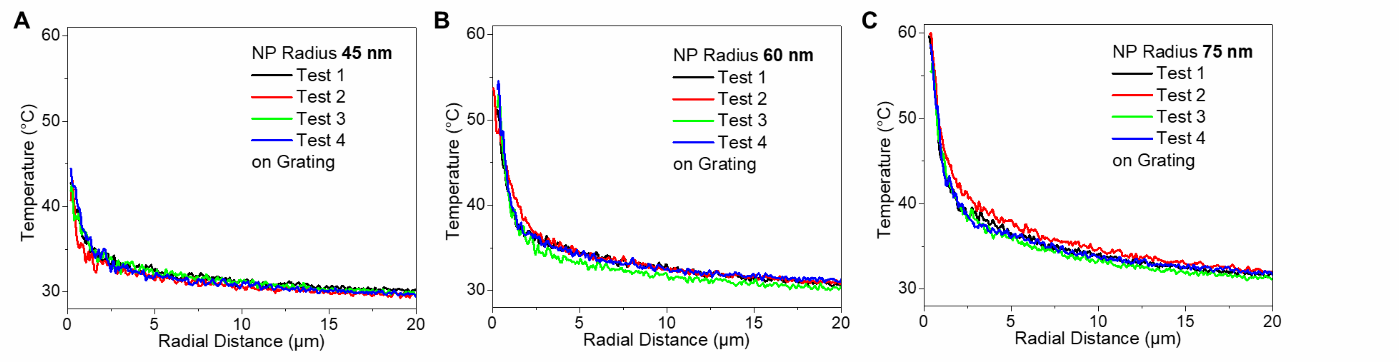

Figure S4. Steady-state radial temperature profiles were obtained experimentally. Four independent heating experiments were conducted for each nanoparticle radius: (A) 45 nm, (B) 60 nm, and (C) 75 nm.
